# Mycobacteriophages and Their Applications

**DOI:** 10.3390/antibiotics13100926

**Published:** 2024-09-27

**Authors:** Andrea Bonacorsi, Caterina Ferretti, Mariagrazia Di Luca, Laura Rindi

**Affiliations:** 1 Department of Biology, University of Pisa, 56126 Pisa, Italy; andrea.bonacorsi@biologia.unipi.it; 2 Department of Translational Research and New Technologies in Medicine and Surgery, University of Pisa, 56126 Pisa, Italy; c.ferretti15@studenti.unipi.it (C.F.); laura.rindi@unipi.it (L.R.)

**Keywords:** mycobacteria, *Mycobacterium smegmatis*, *Mycobacterium abscessus*, *Mycobacterium tuberculosis*, *Mycobacterium avium*, bacteriophages, mycobacteriophages, phage therapy, antibiotic resistance, lung infections

## Abstract

Mycobacterial infections caused by tuberculous and non-tuberculous strains pose significant treatment challenges, especially among immunocompromised patients. Conventional antibiotic therapies often fail due to bacterial resistance, highlighting the need for alternative therapeutic strategies. Mycobacteriophages are emerging as promising candidates for the treatment of mycobacteria. This review comprehensively explores phage isolation, characterization, and clinical applications. Despite the need for more extensive in vitro and in vivo studies, existing evidence shows their efficacy against both sensitive and antibiotic-resistant mycobacterial strains, even under disease-mimicking conditions, particularly when used in cocktails to minimize resistance development. Mycobacteriophages can be engineered and evolved to overcome limitations associated with lysogeny and narrow host range. Furthermore, they exhibit activity in ex vivo and in vivo infection models, successfully targeting mycobacteria residing within macrophages. Delivery methods such as bacterial and liposomal vectors facilitate their entry into human cells. Considering the potential for phage-treatment-induced bacterial resistance, as described in this review, the combination of mycobacteriophages with antibiotics shows efficacy in countering mycobacterial growth, both in the laboratory setting and in animal models. Interestingly, phage-encoded products can potentiate the activity of relevant antibiotics. Finally, the application of phages in different compassionate cases is reported. The positive outcomes indicate that phage therapy represents a promising solution for the treatment of antibiotic-resistant mycobacteria.

## 1. Introduction

Mycobacteria constitute a group of bacteria characterized by a peculiar cell wall rich in mycolic acids, glycolipids, and glycopeptidolipids, which makes them resistant to several antibiotics and gives them distinctive staining properties (acid-fast staining) [1]. *Mycobacterium tuberculosis*, the causative agent of tuberculosis, is the most extensively studied species within this group, representing one of the major causes of death by a single infectious agent worldwide according to the latest WHO report, with an estimated 1.3 million deaths in 2022 [2]. Non-tuberculous mycobacteria (NTM), consisting of all *Mycobacterium* species except for *M. tuberculosis* complex and *Mycobacterium leprae*, are a group of approximately 200 ubiquitous environmental species, generally endowed with low pathogenicity to humans. However, there is a global increase in infections caused by NTM [3]. NTM are classified as either rapidly growing mycobacteria (RGM) or slowly growing mycobacteria (SGM). In particular, *Mycobacterium abscessus* (*M. abscessus* subspecies *abscessus*, *M. abscessus* subspecies *bolletii*, and *M. abscessus* subspecies *massiliense*), *Mycobacterium chelonae*, and *Mycobacterium fortuitum*, as well as the non-pathogenic *Mycobacterium smegmatis*, are part of the RGM group. In contrast, *Mycobacterium avium* complex, *Mycobacterium kansasii*, along with the true pathogen *Mycobacterium ulcerans*, belong to the SGM group. NTM are ubiquitously present in the environment, especially in soil and water, with transmission occurring through the inhalation of contaminated droplets [4]. However, there have been rare occurrences of transmission when exposed to individuals who are infected [5]. Once acquired, NTM infections can lead to pulmonary disease, as well as skin and soft tissue infections and disseminated disease, particularly in individuals with compromised immune systems, structural lung disease, and cystic fibrosis (CF) [6].

**Figure 1:**
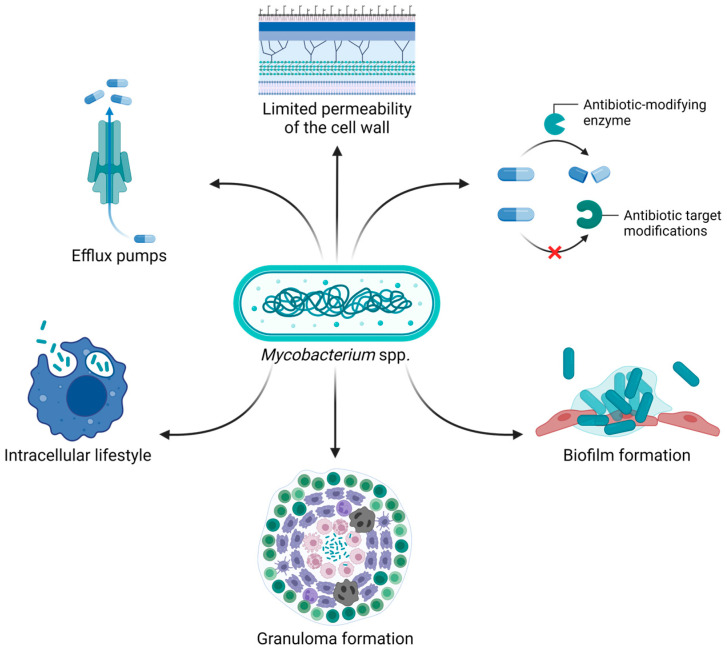
The intrinsic and acquired antibiotic resistance of mycobacteria, combined with their ability to reside within human cells and form granulomas and biofilms, makes these bacteria particularly difficult to treat. Image created with BioRender.com.

Currently, the treatment of mycobacteria primarily involves the administration of different antibiotics, including macrolides, aminoglycosides, β-lactams, rifamycins, and antimycobacterial agents such as isoniazid and ethambutol [7,8]. However, poor outcomes and high mortality rates are common, partly attributed to their intrinsic, acquired, and adaptive antibiotic resistance [9,10]. The mycobacterial cell wall is known to have limited permeability, especially when it comes to antibiotics. In addition, factors such as slow bacterial growth, expression of efflux pumps that recognize antibiotics as substrates, synthesis of antibiotic-inactivating enzymes, and modification of the drug targets contribute to antibiotic resistance [9,10]. Moreover, the ability of mycobacteria to live within human cells (e.g., macrophages) is strictly associated with treatment failure. Indeed, intracellular bacteria are protected from antibiotics that cannot efficiently penetrate the host cell, in addition to being shielded from the host immune system [11]. Biofilm and granuloma formation further increase the challenges in treating infections caused by mycobacteria. In fact, the complex biofilm matrix protects bacteria from penetration of both antibiotics and immune cells. On the other hand, mycobacteria within granulomas can enter a dormant state, making them less susceptible to antibiotic therapy and leading to recurrent infections after reactivation (Figure 1) [6,12].

Given the threat of infections with antibiotic-resistant mycobacteria and the limited progress in the development of new antimicrobials, there is an urgent need to develop alternative therapeutic options. One of the most promising approaches is represented by bacteriophage (phage) therapy, which involves the use of viruses as natural predators of bacteria. The aim of this review is to comprehensively explore mycobacteriophages, examining their in vitro, ex vivo, and in vivo efficacy against different mycobacterial species. From their initial isolation to the ultimate clinical application, this work aims at providing an exhaustive characterization, not only highlighting the challenges encountered at each step, but also describing the proposed strategies to overcome these limitations.

## 2. Mycobacteriophages

Bacteriophages are commonly isolated from environments where bacteria are found. Mycobacteriophages are typically isolated using mostly environmental samples, such as soil, as a source [13,14,15,16,17]. However, other sources may be used, including wastewater samples [18]. Moreover, *M. smegmatis* is employed as a host for phage isolation, which comes with several advantages, including no pathogenicity, fast growth, and shared structural/physiological characteristics with pathogenic and non-tuberculous mycobacteria [19].

In 2010, Hatfull and colleagues created a phage repository named PhagesDB.org [20], aiming at collecting phage data in a systematic way. Out of 26,045 described actinobacteriophages, 13,559 (52%) are mycobacteriophages (September 2024). Most of them (99.6%) were isolated by using *M. smegmatis* mc^2^155 as host, which is generally selected because it is a prophage-free strain, allowing the obtention of phage preparations without the contamination of induced temperate phages [21]. Despite the fact that phages have been isolated on *M. smegmatis*, the analysis of their genomes suggested that, due to the lack of similarities in the DNA GC contents and codon bias usage patterns among many mycobacteriophages, the preferred host of many of them is not *M. smegmatis* [22]. In fact, other strains of *M. abscessus*, *M. avium*, *M. tuberculosis*, *M. phlei*, *M. aurum*, *M. neoaurum*, *M. chelonae*, and *M. aichiense* have also been employed to isolate phages.

The mycobacteriophages characterized so far belong to two different morphotypes, myoviruses and siphoviruses, which are characterized by a double-stranded DNA genome enclosed within a capsid attached to an either contractile or non-contractile long tail, respectively. Interestingly, podoviral mycobacteriophages remain unidentified, likely due to a physical barrier provided by the singular mycobacterial cell envelope [23].

For understanding both the potential clinical applications of phages and their biology, a comprehensive genotypic and phenotypic characterization is needed. In particular, genotypic information is fundamental to exclude the presence of genes associated with antimicrobial resistance, toxins, and virulence factors, thus reducing safety concerns related to phage administration [24]. Furthermore, mycobacteriophages have been categorized into different genomic groups known as clusters (e.g., cluster A, B, C, etc.), which are further subdivided into subclusters (e.g., subcluster A1, A2, A3, etc.) or designated as singletons when there is a lack of close relatives [25]. According to PhagesDB.org, there are currently 34 cluster and 7 singletons (September 2024). This classification is based on shared genes. Phages sharing at least 35% of their genes are grouped within the same cluster [25]. Interestingly, all mycobacteriophages with myoviral morphology belong to cluster C [23], suggesting a limited genetic diversity among this morphology group in comparison to siphoviral mycobacteriophages. In addition, genomic analyses offer insights into phage host preferences since there is a correlation between genome similarity and host range [26,27]. Only phages belonging to clusters G, K, and AB, together with those falling within subclusters A2 and A3, could infect *M. tuberculosis* H37Rv [26,27]. Therefore, phages belonging to these specific clusters/subclusters can potentially be selected and tested in vitro against such a pathogen, serving as a preliminary step towards potential clinical applications.

For phenotypic investigation, the host range determination, which is the range of bacterial species—or, more likely, bacterial strains—being lysed by a single phage is crucial to evaluate the effectiveness of a mycobacteriophage against different mycobacterial species or strains. Phages targeting different mycobacterial species, such as *M. abscessus*, *M. avium*, *M. tuberculosis*, *M. fortuitum*, *M. kansasii*, *M. chelonae*, and *M. ulcerans* have been described [15,16,28,29,30,31]. Although thousands of unique mycobacteriophages have been identified to date, most of them exhibited a narrow host range, only infecting a few strains within a single species. Hatfull et al. demonstrated the extensive genomic diversity among mycobacteriophages, suggesting that this diversity could potentially provide broad coverage across various mycobacterial strains [20]. Nevertheless, achieving complete coverage would likely necessitate a large and diverse library of mycobacteriophages representing a wide array of host specificities. Even if it is rather rare, a few mycobacteriophages with a broad host range have been reported, being effective in killing both tuberculous and NTM species [16,30,31]. For instance, phages G1, J1, and D1 were found to simultaneously infect *M. tuberculosis*, *M. avium*, *M. fortuitum*, and *M. kansasii* [16], while phages Bxz2, D29, and L5 exhibited lytic activity against *M. tuberculosis*, *M. avium*, *M. ulcerans*, *M. fortuitum*, and *M. chelonae* strains [30]. The ability of certain mycobacteriophages to infect both slowly and rapidly growing mycobacteria highlights their adaptation to different bacterial life cycles.

Based on phage characterization, most of the mycobacteriophages showed a latent period ranging between 30 and 135 min and a burst size of approximately 100–200 new virions produced from a single infection event [13,14,15,17,32]. In addition, phages of mycobacteria appear to be more stable at alkaline pH values and are inactivated at pH values equal to or lower than 5 and at high temperatures, such as 55 °C or higher [15,17].

Kalapala and colleagues demonstrated that a phage cocktail comprising five phages (D29, TM4, Che7, PDRPv, and PDRPxv) resulted in activity against *M. smegmatis* mc^2^155 at 5.5–6 pH conditions [33]. Low-pH conditions (values of 4–6) are typically observed in infected phagosomes [34]. The cocktail also exhibited activity against *M. smegmatis* mc^2^155 in hypoxic environments, such as those found within granulomas, as well as against its non-replicative bacterial form during the stationary phase [33]. These results suggest a potential application in in vivo infection conditions. Interestingly, the five-phage cocktail prevented the emergence of bacterial clones resistant to phages. In addition, phages were also found to be active against isoniazid-resistant *M. smegmatis* mc^2^155 [33]. Finally, two phages of the cocktail (D29 and TM4) were combined with another phage (DS6A), showing the ability to lyse *M. tuberculosis* H37Ra during both lag and exponential phases of growth, preventing bacterial regrowth for several weeks (up to 57 days). In contrast, a different three-phage cocktail (D29, TM4, and Che7) at the same multiplicity of infection (ten phages per bacterial cell) showed bacterial regrowth after just 4 days when tested against *M. smegmatis* mc^2^155 [33]. Although the two three-phage cocktails differ by only one phage, making their comparison less than ideal, the results highlight significant differences in the control of bacterial growth between slowly and rapidly growing mycobacteria.

## 3. Activity of Mycobacteriophages Ex Vivo and In Vivo

As mentioned above, intracellular mycobacteria are protected from both the host immune system and antibiotic therapy, potentially resulting in treatment failure [11]. Therefore, it is crucial to investigate the ability of mycobacteriophages to reach bacteria residing within human cells, particularly macrophages, considering that assessing phage stability and activity in low-pH environments is essential before conducting the experiments.

Phages are known to be endocytosed by human cells, primarily through a non-specific macropinocytosis and, to a lesser extent, receptor-mediated phagocytosis. Indeed, both endocytic pathways can engulf objects within the micrometer range, allowing phage uptake. In the context of infections, phagocytosis is driven by the recognition of specific ligands, such as pathogen-associated molecular patterns (PAMPs), which bind to human cell receptors known as pattern-recognition receptors (PRRs). However, no specific PAMPs on phage surfaces that interact with surface-exposed PRRs have been identified to date. Consequently, it is more likely that phages, after lysing bacterial cells, interact with PAMPs deriving from bacterial debris, potentially triggering phagocytosis. Furthermore, phagocytosis may be facilitated by opsonic receptors (i.e., antibodies and complement proteins) [35]. Although phages can enter human cells, their activity within them remains to be fully understood.

Initial efforts to target intracellular mycobacteria with phages involved administering the non-pathogenic *M. smegmatis* mc^2^155 infected with the lytic phage TM4. The infected strain acted as a “Trojan horse” to deliver phages within RAW 264.7 mouse macrophages infected with either *M. avium* or *M. tuberculosis*. The results demonstrated a significant reduction (up to 100-fold) in the viability of both intracellular *M. avium* and *M. tuberculosis* over time in comparison to an untreated control and the administration of uninfected *M. smegmatis* mc^2^155. Importantly, phage treatment alone was not able to kill intracellular mycobacteria, even at higher phage titers (10^7^ PFU). In addition, both time-lapse video and fluorescent microscopy showed that the vacuole containing phage-infected *M. smegmatis* fused with the vacuole harboring *M. avium* [36].

Confirmation of these findings occurred in vivo using mice infected with *M. avium* [37]. Indeed, mice treated with *M. smegmatis* mc^2^155 carrying TM4 exhibited a significant decrease (0.5 logs) in intracellular bacteria at the level of the spleen compared to untreated control mice, as well as compared to mice treated with only TM4 phages or only *M. smegmatis* mc^2^155 without TM4, where no reduction was observed. Interestingly, no further decrease in bacterial viability was noted when the treatment was administered twice. In fact, a modest fraction of bacteria recovered from treated mice showed resistance to the phage. However, the lower treatment efficacy may also be attributed to the inability of the bacterial vehicle to effectively deliver the phage to all mycobacterial vacuoles [37].

Potential risks associated with the administration of the mycobacterial vector include an excessive presentation of bacterial antigens to the host immune system, which could trigger an immune response against the vector, reducing its effectiveness. In addition, the vector might acquire virulence genes from the pathogenic bacterium. To address these concerns, a second attempt to target intracellular mycobacteria involved using non-bacterial vectors to deliver phages into infected eukaryotic cells. For instance, phages can be encapsulated within liposomes. Specifically, the TM4 phage and the *Escherichia coli* λeyfp phage were encapsulated into giant liposomes using different techniques. Although the encapsulation efficiency may be low, λeyfp phages interacting externally with liposomes were more efficiently taken up into THP-1 human macrophages compared to free phages, localizing in the endocytic compartments [38]. Even though the investigation of human cell uptake was limited to the *E. coli* phage, these findings suggest that TM4 mycobacteriophages might act similarly, finally localizing where mycobacteria reside [38]. The significantly low uptake of free phages observed in the THP-1 cells is consistent with the phage treatment failure showed in infected mouse macrophages reported above [36]. In another study, Lapenkova et al. encapsulated D29 mycobacteriophage within liposomes and evaluated its efficacy against intracellular *M. tuberculosis.* By targeting infected RAW 264.7 macrophages and tuberculous granuloma with phage liposomes, they showed a higher bactericidal activity compared to free phages [39].

More recently, widefield fluorescence microscopy and 3D deconvolution were employed to demonstrate the uptake of three different phages by THP-1 macrophages, primary murine bone marrow-derived macrophages (BMDM), and A549 lung epithelial cells. The percentage of cells exhibiting intracellular phages was very variable, ranging from 7 to 90% depending on cell type, phage type, and phage titers. For this reason, differences in treatment outcomes are expected. In addition, two out of the three phages (singularly administered) significantly reduced intracellular *M. abscessus* viability. In particular, this occurred within A549 cells when phages were administered at high concentrations, resulting in reductions ranging from 0.3 to 2.1 logs. However, the inefficacy of one phage in killing intracellular mycobacteria highlights that not all phages may act effectively in intracellular environments. Finally, fluorescence and transmission electron microscopy further demonstrated the co-localization of both phages and mycobacteria within human cells [40]. The discrepancy in the killing mediated by free phages on intracellular *M. abscessus*, compared to TM4 treatment of intracellular *M. avium* and *M. tuberculosis* [36], may be attributed to differences in cell types, bacteria, phages, and phage concentrations, rather than phage size. Thus, further research is needed to understand the interplay between these factors when assessing phage efficacy against intracellular mycobacteria.

## 4. Bacteriophage-Resistant Mycobacteria

Similarly to antibiotics, phages exert a strong selective pressure on their bacterial host, driving the evolution of resistance. This can lead to the emergence of bacteria resistant to phages, potentially resulting in therapy failure. Various mechanisms contribute to phage resistance, including the inhibition of phage adsorption as well as the prevention of phage genome injection, replication, and assembly of new viral particles [41]. Specifically, mutation or downregulation of the bacterial receptor can impair phage adsorption [41]. Furthermore, receptors can be masked by extracellular polymeric substances or masking proteins. Innate and adaptive bacterial immunity can play a role in phage resistance. Indeed, the restriction–modification and the CRISPR–Cas systems can contribute to the degradation of the phage genome. Beyond protection at the individual cell level, infected bacteria can activate the abortive infection system upon recognition of phage-specific components, leading to bacterial cell death and thereby protecting uninfected cells [41].

**Figure 2:**
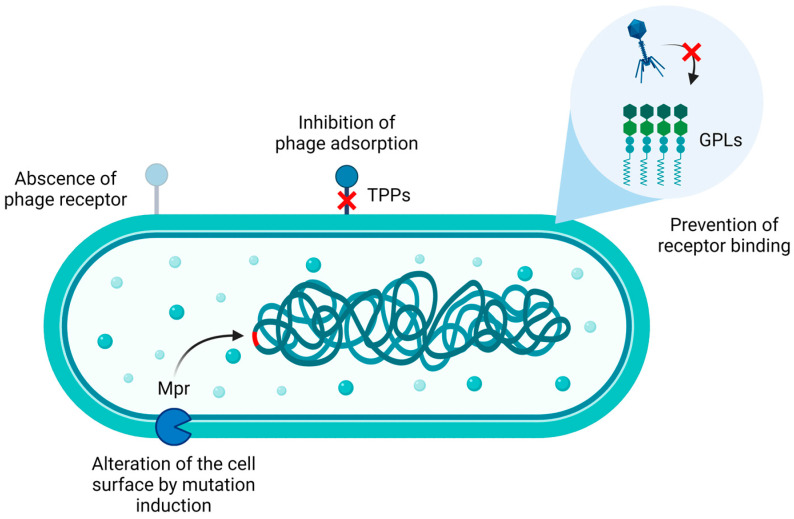
Phage resistance mechanisms in *M. abscessus* and *M. smegmatis* may involve the absence of phage receptors, mutations in phage receptors/co-receptors such as trehalose polyphleates (TPPs) [43], the presence of glycopeptidolipids (GPLs) [44,45], and the induction of mutations in the bacterial genome, eventually altering its surface and impairing phage adsorption, as in the case of the exonuclease Mpr [46,47]. Image created with BioRender.com.

To date, according to the literature, mycobacterial phage resistance has been mainly explored in *M. abscessus* and *M. smegmatis* (Figure 2). In particular, *M. abscessus* phage resistance appears to be primarily related to the colony morphotype. Indeed, the microorganism generates either smooth (S) or rough (R) colonies when grown on solid medium. The S phenotype is characterized by the presence of glycopeptidolipids (GPLs) on the bacterial surface, which are recognized by the host immune system, resulting in decreased virulence compared to their rough counterparts. On the other hand, the R phenotype is associated with the impairment of either GPLs biosynthesis or their transport to the bacterial envelope. Given that R strains can escape the immune system, they are considered more virulent [42].

In experiments involving eight phages tested for their bactericidal activity against several *M. abscessus* clinical isolates, a small fraction of the S strains (21%) was lysed by a few phages when tested for plaque formation, although none of them was killed when challenged in liquid medium. On the other hand, most of the R strains (80%) were efficiently killed by at least one of the small panel of phages, as assessed by both plaque formation and liquid infection assay [44]. These results were further validated using an additional large set of *M. abscessus* clinical isolates, which were examined for their sensitivity to eleven phages. The findings revealed that a higher percentage of R strains (77%) were successfully infected compared to S strains (48%), as determined by the plaque formation assay. Similarly, when challenged in liquid culture, S strains did not exhibit efficient lysis [45]. As a consequence, GPLs expressed on the surface of S strains may be responsible for the lower phage infection properties [44,45].

Mycobacteria may also be intrinsically resistant to mycobacteriophage infections. For instance, they may not express the receptor recognized by a specific phage as well as they can physiologically synthetize GPLs. Although bacteria may be susceptible to phage infections, mutations conferring resistance to phages could occur. In this context, when strains susceptible to the previous panel of eight phages were subjected to phage challenges for the isolation of phage-resistant mutants, the obtained mutants were sequenced and compared to their respective parental strains. The results showed that acquired phage resistance could be associated with reversion events to the smooth phenotype. However, this happens rarely because the R phenotype commonly results from indels in the GPLs biosynthesis genes *mps1* and *mps2*, thus decreasing the probability of reversion to the S phenotype. Furthermore, mutations in genes involved in trehalose polyphleates (TPPs) biosynthesis, as well as alterations in virulence genes and, possibly, plasmid loss, can confer bacterial resistance to phages [44].

In particular, TPPs are necessary for infection of *M. abscessus* and *M. smegmatis* by BPs and Muddy phages. Mutations in these surface-exposed glycolipids resulted in adsorption impairment, thereby conferring phage resistance. However, single amino acid substitutions in the tail spike proteins enabled phages to infect their host, suggesting that TPPs may act as co-receptors [43].

Finally, evidence suggests that the non-specific, membrane-bound DNA exonuclease Mpr could be involved in mycobacteria phage resistance [46,47]. Initially, an attempt to elucidate its involvement in bacterial resistance involved the overexpression of Mpr in *M. smegmatis*, resulting in bacterial resistance to two phages, which could be associated to the inhibition of the ejection of their genetic material into bacterial cells. However, this hypothesis is unlikely because of cytotoxic effects associated with Mpr overexpression [47]. Further investigations suggested that, more probably, Mpr may contribute to phage resistance by either interacting with bacterial DNA (directly or indirectly) or activating downstream pathways such as stress response pathways, ultimately leading to mutations in the bacterial genome. These mutations may increase bacterial survival against phage infection by altering the cell surface [46].

**Figure 3:**
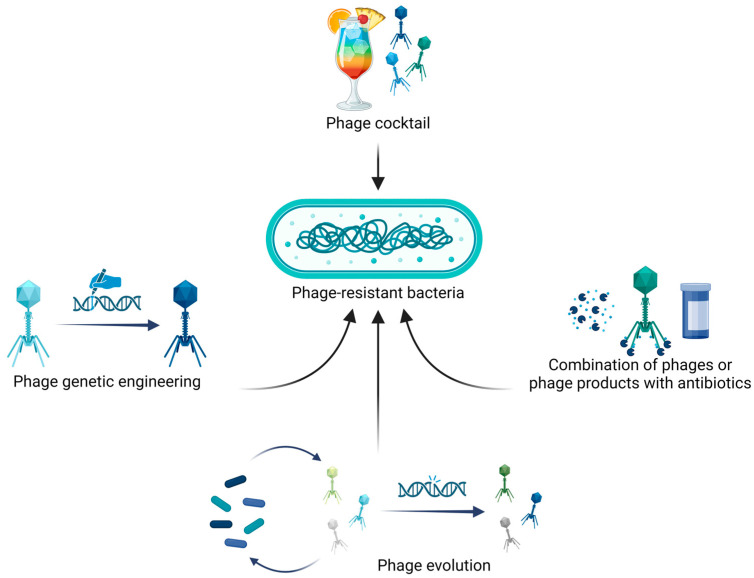
Phage therapy strategies to counter bacterial resistance include the combination of multiple highly efficient phages into phage cocktails; phage genetic engineering to remove genes involved in lysogeny, antibiotic resistance or virulence factors; phage evolution, which allows the obtention of phages with higher killing efficacies and expanded host ranges; and, finally, the combination of phages or phage-derived products (e.g., lysins) with antibiotics to increase bactericidal activity. Image created with BioRender.com.

Although bacterial resistance to phages might be a limit in their applications, different approaches have been developed to overcome such phenomenon, as indicated in Figure 3 and reported in the next paragraphs.

## 5. Combination of Phages and Phage-Encoded Products with Antibiotics

To improve treatment efficacy and address potential phage resistance, phages can be combined with antibiotics. This combination not only increases bactericidal activity but also allows the reduction of antibiotic doses, thereby minimizing adverse effects on the normal microbiota. Considering the promising antimicrobial activity previously observed in phage–antibiotic combinations, investigation of their potential synergistic activity is crucial [24]. For instance, a five-phage cocktail demonstrated in vitro synergy with rifampicin and isoniazid against *M. smegmatis* mc^2^155 and an isoniazid-resistant *M. smegmatis* mc^2^155 strain, respectively [33]. In addition, phage Muddy showed an adjuvant effect with rifabutin, imipenem, bedaquiline, clofazimine, tigecycline, and amikacin but not with clarithromycin and linezolid when tested against *M. abscessus*. Importantly, this cooperative activity was demonstrated in vivo using an infection model of CF zebrafish, showing an increase in larval survival rates compared to single treatments, accompanied by a decrease in symptoms [48]. Moreover, the tuberculocidal activity of a phage combined with either isoniazid or rifampicin was tested against *M. tuberculosis*. In this case, although the experimental conditions did not allow the determination of whether the combination was synergistic, no antagonism between the phage and the antibiotics was observed, suggesting their compatibility for a potential clinical application [27]. In contrast, Jiang and colleagues showed that two phages could not infect *M. tuberculosis* in the presence of the aminoglycoside antibiotics kanamycin, hygromycin, or streptomycin due to inhibition of phage DNA replication. Antagonism was not observed for another aminoglycoside, spectinomycin. Considering that spectinomycin does not contain an amino sugar group compared to the other tested antibiotics, this functional group might be involved in blocking phage DNA synthesis. Remarkably, these findings suggest that *M. tuberculosis* strains susceptible to only kanamycin, hygromycin, or streptomycin might preclude the use of concomitant phage therapy with the tested phages when in vitro antagonism is observed [49].

In addition to the direct use of phages, either alone or in combination with antibiotics, phage-encoded products can be used as therapeutic agents. Mycobacteriophages lysins, for instance, can degrade the complex mycobacterial cell wall. In this context, lysin A (LysA) is a peptidoglycan hydrolase that cleaves specific bonds within the peptidoglycan layer, while lysin B (LysB) is an esterase that cleaves the linkage between mycolic acid and arabinogalactan. Consequently, this combined action results in osmotic lysis of the bacterial cell [50]. An example of lysin with therapeutic potential is LysB synthetized by the lytic D29 phage. This lysin efficiently lysis both drug-sensitive and drug-resistant *M. tuberculosis* strains, exhibiting additive activity with rifampicin. In addition, after showing no cytotoxicity, LysB was demonstrated to be active against RAW 264.7 mouse macrophages infected with this bacterium, especially when administered with a combination of isoniazid and rifampicin [51]. PK34 is another D29 phage-derived product possessing tuberculocidal activity [52]. Besides lysins, other phage proteins can be employed as therapeutics. For example, two proteins from the SWU1 phage, namely gp36 and gp67, demonstrated the ability to potentiate antibiotic activity [53,54]. Specifically, when the gp39 protein of the phage was overexpressed in *M. smegmatis* mc^2^155, it downregulated genes associated with cell wall and biofilm formation. In particular, this protein affected the lipid metabolism of the bacterium, increasing the permeability of the bacterial envelope. As a result, it potentiated the efficacy of antibiotics such as isoniazid, erythromycin, norfloxacin, ampicillin, ciprofloxacin, ofloxacin, rifampicin, and vancomycin. Furthermore, it enhanced susceptibility to some environmental stresses, including hydrogen peroxide, heat shock, low pH, and surfactants [53]. Similarly, overexpression of gp67 in *M. smegmatis* mc^2^155 resulted in both colony and biofilm alterations, along with increased susceptibility to streptomycin and capreomycin. Also in this case, gp67 overexpression downregulated genes involved in cell envelope and biofilm development [54]. Consequently, gp36 and gp67 might be used as adjuvant in combination with antibiotics.

## 6. Mycobacteriophage Engineering and Evolution

**Figure 4:**
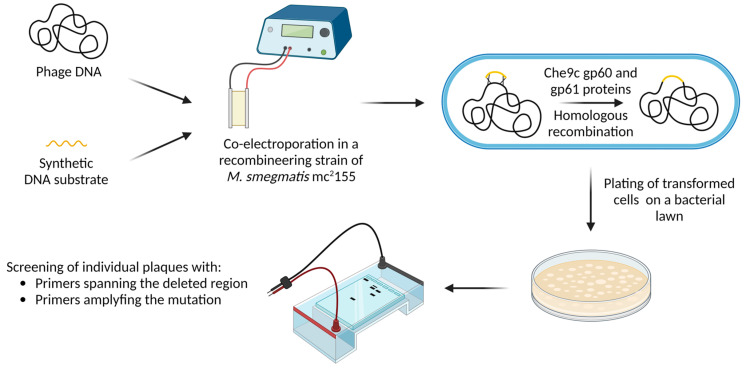
Overview of the bacteriophage recombineering of electroporated DNA (BRED) technique. Phage and synthetic DNA are co-electroporated into a *M. smegmatis* mc^2^155 strain carrying a plasmid encoding homologous recombination proteins (gp60 and gp61). Homologous recombination between phage and synthetic DNA, with subsequent plating of transformed bacteria, allows the obtention and screening of plaques to identify deletion mutants [56]. Image created with BioRender.com.

Most of the newly isolated mycobacteriophages are temperate, making them unsuitable for clinical applications due to their ability to integrate their nucleic acids into the host genome as prophages. In this context, integrated phages slow down bactericidal activity and increase the possibility of antibiotic resistance or virulence genes being transferred from one bacterial cell to another by transduction [21]. However, temperate phages or prophages spontaneously released from mycobacteria might be a source of viruses which can be isolated and genetically engineered to be strictly lytic, overcoming this limitation. One example is phage ZoeJ, in which gene *45*—essential for lysogeny—was deleted using the bacteriophage recombineering of electroporated DNA (BRED) methodology, conferring a strictly lytic phenotype [55]. BRED is a technique that enables the obtention of marker-less, in-frame gene deletions, as well as base substitution, the addition of gene tags, and the insertion of foreign genes [56]. This can be achieved by employing a recombineering strain of *M. smegmatis* mc^2^155. Indeed, the ectopic expression of two proteins from the mycobacteriophage Che9c, namely gp60 and gp61 (an exonuclease and a DNA-binding protein, respectively), confers high levels of homologous recombination in this strain [57]. In the BRED protocol, phage genomic DNA and a synthetic DNA substrate containing sequences flanking the gene to be deleted are co-electroporated into the recombineering strain of *M. smegmatis* mc^2^155, allowing gene deletion through homologous recombination. Transformed bacterial cells are plated on a bacterial lawn and, after incubation, individual plaques are screened with primers spanning the deleting region and/or primers selectively amplifying the mutation, allowing discrimination between wild-type and mutant phages. Generally, plaques containing both phage types are initially recovered. Subsequently, these mixed plaques are plated again, and individual plaques are screened with the same set of primers until pure mutant plaques are obtained (Figure 4) [56]. Interestingly, BRED can be combined with CRISPR-Cas9 systems to counter-select against the wild-type phage, enabling the enrichment of mutants. This approach is particularly relevant when recombination efficiency is low [43]. Importantly, phage engineering can be useful for deleting antibiotic-resistance genes or bacterial-virulence factors, as well as expanding the phage host range and even arming the phage with additional antimicrobial capabilities [58].

Phages isolated using *M. smegmatis* as host might be unable to efficiently infect pathogenic mycobacteria. To overcome this limitation, one strategy involves isolating host range mutants, which are phages that gain the ability to effectively infect the specific bacterium of interest. For instance, Jacobs-Sera and colleagues obtained a limited number of plaques when testing two phages against *M. tuberculosis* mc^2^7000 lawns, which was due to the reduced efficiency of phage infection. These plaques were isolated and subsequently re-plated on both *M. tuberculosis* mc^2^7000 and *M. smegmatis* mc^2^155 lawns, achieving an equal efficiency of plating. Purified plaques from the *M. smegmatis* mc^2^155 plate were then confirmed to infect both strains with identical efficiency of plating [26]. Not surprisingly, the mutated genes were those associated with the phage tail [26]. To increase the infectivity and lytic activity of mycobacteriophages, they can also be adapted to their hosts through serial passages under different conditions [59]. These conditions include different phage inoculum loads (small or large) and the use of either liquid or semi-solid media. Indeed, phage bactericidal activity against *M. smegmatis* mc^2^155 increased with an escalating number of serial passages in the presence of the phages, especially under small-inoculum conditions [59]. Furthermore, phage cocktails can be evolved using the Appelmans method [60]. This technique involves culturing phage cocktails on different bacterial strains, with most of them being resistant to phage treatment. Through repeated cycles of evolution, phages may overcome resistance, eventually broadening their host range [60]. Employing this protocol, sixteen mycobacteriophages exhibiting killing activity against *M. abscessus* underwent directed evolution. As a result, after only eleven of thirty rounds of evolution, they gained the ability to target clinical strains previously resistant to unevolved phages [18].

Engineered and evolved phages can be assembled into phage cocktails, along with natural wild-type phages. In this context, a cocktail constituted of five phages selected among those engineered and evolved phages demonstrated bactericidal activity against both isoniazid-sensitive and isoniazid-resistant *M. tuberculosis*, also minimizing the emergence of phage resistance [27].

## 7. Therapeutic Application of Mycobacteriophages

Over the past 20–25 years, there has been an increase in published case reports and clinical trials regarding phage therapy. Therapeutic applications of bacteriophages mainly involve pulmonary infection and implantable medical device-related infections, as well as urinary tract infections, primarily caused by *Pseudomonas aeruginosa*, *Klebsiella pneumoniae*, and *Staphylococcus aureus* [61,62,63]. However, case reports and clinical trials regarding the application of phage therapy for the treatment of infections caused by mycobacteria are limited. Case reports published to date are summarized in Table 1.

The first clinical case reporting treatment based on mycobacteriophages was a 15-year-old patient with CF [66]. Seven months after lung transplantation, the patient was diagnosed with disseminated *M. abscessus* subspecies *massiliense* infection, associated with two skin lesions developed on the forearm and granulomatous inflammation at a sternal wound. Three phages, named Muddy, BPs33ΔHTH-HRM10, and ZoeJΔ45, were administered. Particularly, Muddy was a natural, strictly lytic, phage, while the two remaining were temperate and genetically engineered to be strictly lytic phages. In addition, phage BPs33ΔHTH-HRM10 is a host range mutant of BPs, isolated to improve the bactericidal activity. The patient was initially treated with a topical test application of the three-phage cocktail on a sternal lesion. After 24 h, the individual was treated intravenously (10^9^ PFU/dose of each phage) every 12 h for at least 32 weeks, with concomitant multidrug antibiotic therapy. After one month, the sternal lesion had improved compared to the other skin wound. Consequently, topical daily phage therapy was administered for the two lesions. Phage treatment was safe and well tolerated by the patient, with no occurrence of adverse effects. The therapy resulted in clinical improvement, with gradual healing of the surgical wound and skin lesions and improved pulmonary function. Microbiological investigations showed that *M. abscessus* was not isolated from serum or sputum at any time after the start of phage therapy, but it was cultured from swabs of slow-resolving skin nodules at 1, 3, 4, and 5 months. After 121 days of treatment, *M. abscessus* isolated from the patient remained sensitive to the three phages in the cocktail, confirming the absence of phage resistance. Here, phage-neutralizing antibody production was not observed. Interestingly, this was also the first clinical case of a patient treated with genetically modified bacteriophages [66].

A more recent case report described a different outcome after intravenous treatment with the same three-phage cocktail at the same dose [67]. The patient, an 81-year-old immunocompetent individual with non-CF bronchiectasis and refractory *M. abscessus* subspecies *massiliense* lung disease, reported no side effects after the administration of mycobacteriophages. However, two months after the start of treatment, a strong immune response developed, resulting in neutralizing activity of the phages. No post-treatment isolates were resistant to all three phages, suggesting that the neutralizing activity of antibodies developed against phages caused the therapeutic failure [67]. After six months of intravenous treatment, the patient received the same phage cocktail at the same dose twice daily by aerosolized delivery, trying to bypass serum neutralization and to enhance phage delivery to the infection site [68]. After 3 months of treatment, the patient’s clinical condition improved. A quantitative reduction of *M. abscessus* in sputum was observed. Unfortunately, after 4 months from the start of nebulized phage administration, transient enhancements disappeared, with consequent clinical and microbiological decline in the patient. Nebulized treatment failure was not attributed to the development of phage resistance, since the isolated strain remained susceptible to all phages, or even to the immune response, as only mild neutralization was noticed. However, the authors believe that the neutralization, increased at later times (7 and 8 months) after the initiation of nebulization, may have contributed to the limitation of the treatment effect. The reasons behind the limited duration of phage aerosolization improvements in this patient remain unclear [68].

Immune reactions and the development of antibodies against bacteriophages pose a serious problem in phage therapy, very often leading to therapeutic failures. Sometimes, however, the development of a robust immune reaction by the host does not compromise phage therapy. In 2022, two patients were successfully treated with mycobacteriophages despite the development of phage-neutralizing antibodies. There is certainly a need to better understand and study incidence, timing, and specificity of immune reactions [70].

Nick and colleagues described the case of a 26-year-old individual with CF and bronchiectasis, as well as *M. abscessus* subspecies *abscessus* lung infection [64]. The patient received a phage cocktail intravenously twice daily for 18 months. The cocktail included BPs33ΔHTH-HRM10 (10^9^ PFU/mL) and D29_HRM^GD40^ (10^8^ PFU/mL). Both are host range mutants of lytic phages. Treatment with mycobacteriophages had no adverse effects, and the development of phage resistance was not observed during therapy. Immunologically, only D29_HRM^GD40^ was active throughout the treatment, while neutralizing antibodies were produced against BPs33ΔHTH-HRM10. Nevertheless, phage therapy, in combination with antibiotics, eradicated the *M. abscessus* infection, making lung transplantation possible for the patient [64].

The first case of *M. chelonae* infection treated with bacteriophage therapy was reported by Little and colleagues [69]. The patient was a 56-year-old man with a refractory disseminated cutaneous infection characterized by nodular lesions on the left upper extremity with spontaneous drainage. Muddy phage was injected intravenously twice a day for more than 6 months at a dose of 10^9^ PFU/dose, in association with an antibiotic treatment. A few hours after the therapy administration, the patient reported nausea and chills, but these resolved spontaneously. No phage resistance was observed during the treatment. The patient developed phage-neutralizing antibodies after 17 days of therapy, which increased after 16 weeks of treatment. Nevertheless, the immune response was not associated with treatment failure. In contrast, signs of clinical improvement were evident in the patient, whose skin lesions showed improvement and reduction in inflammation and nodules. The infection was successfully eradicated [69].

Recently, a case series on the compassionate use of mycobacteriophages was reported [65]. Twenty patients involved in the study had mycobacterial infections difficult to treat with antibiotics, mainly caused by *M. abscessus*, *M. chelonae*, *M. avium*, and BCG. They received phages at a dose of 10^9^ PFU twice a day intravenously, by inhalational nebulization, or both, in conjunction with an antibiotic regimen. A positive clinical and microbiological outcome was observed for 11 patients. In particular, five patients had a favorable outcome, while for the other 6 patients, the outcome was partially favorable, mainly due to complications from other infections. Four patients had no responses to therapy, and five patients had inconclusive responses. Phage therapy was well tolerated by all patients, who showed no serious side effects. In addition, the development of phage resistance was not observed, even in those patients treated with a single phage and not with a cocktail. Host immune reactions were observed in ten patients, with one of them being characterized by a weak neutralizing antibody response, however without correlation between neutralization and outcomes [65].

Treatment of antibiotic refractory NTM infections with phage therapy seems to be a promising solution. These few cases have shown the safety of phage treatment [71]. Development of phage resistance is possible but appears to be infrequent, even when a single phage is administered [65]. In most cases of failure of phage therapy, induction of neutralizing antibodies is the major cause. One possible strategy to prevent this problem could be the encapsulation of phages in liposomes, as reported by in vitro and in vivo studies [38,72]. In this way, phages are not targeted by neutralizing antibodies. In addition, as mentioned earlier, the poor ability of bacteriophages to penetrate eukaryotic cells could be enhanced through this system, which allows phages to enter intracellularly, such as in macrophages, where most mycobacteria replicate [38,65]. The pharmacodynamics and tissue penetration of the phages still need to be fully explored and investigated to define the optimal route of administration and dosage. Intravenous administration is desirable for the treatment of disseminated infections and could be a valid option in the case of infections characterized by structural lung damage (fibrosis or bronchiectasis) or when mucoid obstruction is present, compromising administration by nebulization alone. Nebulization, meanwhile, could also avoid systemic neutralization [65]. However, if aerosol delivery is chosen as the administration route, phage stability after nebulization needs to be assessed [73].

Interestingly, individuals with CF commonly suffer from polymicrobial infections. Indeed, they may present NTM infections while being chronically infected with *P. aeruginosa* and/or *S. aureus* [53,62], as well as *Haemophilus influenzae*, *Stenotrophomonas maltophilia*, and *Burkholderia* species [74]. The coexistence of multiple bacterial species in the airways could exacerbate the clinical condition and accelerate disease progression in CF patients. Campo-Pérez and colleagues demonstrated that coinfection with *M. abscessus* and *P. aeruginosa* led to a more rapid reduction in the viability of *Galleria mellonella* larvae compared to infection with *P. aeruginosa* alone [75]. Additionally, in advanced CF lung disease, the antibiotic regimen for treating *M. abscessus* infections often fails due to bacterial resistance to various drug classes such as aminoglycosides, rifamycins, tetracyclines, and β-lactams [76]. Moreover, bacterial infections cause lung structural damage, such as cavities and regions of parenchymal collapse, which may hinder the penetration of antibiotics into the affected regions [62]. For these reasons, CF patients with coinfections are usually treated with prolonged antibiotic therapies, which can promote the selection of resistant bacterial clones, as well as antibiotic toxicity [76]. To curb these issues, one potential therapeutic approach could be the use of a phage cocktail containing bacteriophages active against the different pathogenic species infecting the CF airways. This strategy might reduce treatment duration and toxicity associated with extended antibiotic use accompanied by the simultaneous treatment of different bacterial infections, while also lowering the inflammatory response.

## 8. Concluding Remarks

The increasing prevalence of multidrug-resistant tuberculous and non-tuberculous mycobacterial infections presents a growing challenge in terms of treatment. However, phage therapy represents a promising alternative strategy. Through in vitro, ex vivo, and in vivo investigations, including various compassionate cases, it has been showed that natural, engineered, and evolved phages can effectively alleviate the burden caused by mycobacteria, especially when used in combination with antibiotics. In fact, it is unlikely that phage therapy will completely replace conventional antibiotic treatment, but it may significantly contribute to addressing infections, particularly when phage cocktails are administered.

It is important to emphasize the necessity of a large, diverse, and highly effective phage library to cover most of the mycobacterial strains. Mycobacteriophages, with their broader host range compared to other phages, exhibit potential activity against several mycobacterial species. However, clinical isolates may display significant variations in phage susceptibility, as observed in the case of *M. abscessus* [44,45], potentially requiring a personalized approach. Interestingly, for *M. tuberculosis*, the clinical isolates seem to share more similarities in terms of phage infection profiles compared to NTM, possibly obviating the need for personalized medicine [27].

Further genotypic and phenotypic analyses are crucial to deepen our understanding of mycobacteriophage activity, such as that against biofilm-embedded mycobacteria. Moreover, the isolation of phage-resistant bacterial strains could be useful to evaluate possible trade-offs. For instance, determining whether the onset of phage resistance restores susceptibility to antibiotics could broaden antibiotic treatment options.

Despite the efficacy demonstrated in compassionate cases of mycobacteriophage therapy against *M. abscessus*, clinical trials involving large cohorts are essential to gain a clearer understanding of this alternative approach, including its safety, pharmacokinetics, pharmacodynamics, immune response, and potential development of bacterial resistance to phages. In particular, to improve the therapeutic approach, it would be fundamental to better comprehend phage delivery, administration route, and dosing. This may become a reality soon, considering the recently initiated clinical trials of phage therapy against *P. aeruginosa* infections in CF individuals. However, infections caused by other mycobacterial pathogens need to be addressed, starting from pre-clinical and compassionate investigations.

## Figures and Tables

**Table 1:** Current clinical cases of therapeutic application of mycobacteriophages.

Infection	Number of Cases	Type of Infection	Underlying Condition	Monophage\Cocktail (Number of Cases)	Administration Route	Antibiotic Combination	Outcome (Number of Cases)	Ref.
*M. abscessus* subsp. *abscessus*	12	Disseminated, lung	CF, lung transplant	Monophage (6), Cocktail (6)	Intravenous, aerosol, bronchoscopic administration	At least 2 drugs for patient	Positive (6), inconclusive responses (2), no response to therapy (4)	[64,65]
*M. abscessus* subsp. *massiliense*	5	Disseminated, lung, sternal bone infection	CF, lung transplant, scleroderma, chronic lung bronchiectasis	Monophage (3), Cocktail (2)	Intravenous, aerosol, topical, chest wash	At least 2 drugs for patient	Positive (2), inconclusive responses (3)	[65,66,67,68]
*M. chelonae*	1	Cutaneous	Seronegative arthritis on immunosuppression	Monophage	Intravenous	Omadacycline, Bedaquiline, and Trimethoprim-sulfamethoxazole	Positive	[65,69]
*M. avium* complex	1	Lung	CF	Monophage	Intravenous, aerosol	At least 2 drugs for patient	Positive	[65]
BCG	1	Disseminated	Mendelian susceptibility to mycobacterial disease, heterozygous mutation in *NFKBIA* gene	Cocktail	Intravenous	At least 2 drugs for patient	Positive	[65]
